# *In vitro* Hypolipidemic and Antioxidant Effects of Leaf and Root Extracts of *Taraxacum Officinale*

**DOI:** 10.3390/medsci3020038

**Published:** 2015-06-11

**Authors:** Belén García-Carrasco, Raquel Fernandez-Dacosta, Alberto Dávalos, José M. Ordovás, Arantxa Rodriguez-Casado

**Affiliations:** 1 IMDEA Food Institute, C/Carretera de Cantoblanco 8, 28049 Madrid, Spain; E-Mails: belen.garcia@imdea.org (B.G.-C.); raquelfernandezdacosta@gmail.com (R.F.-D.); alberto.davalos@imdea.org (A.D.); jose.ordovas@tufts.edu (J.M.O.); 2 Nutrition and Genomics Laboratory, JM-USDA Human Nutrition Research Center on Aging at Tufts University, Boston, MA 02111-1524, USA

**Keywords:** adipocyte, antioxidant, cholesterol, dandelion, polyphenols, triglycerides

## Abstract

Adipose tissue dysfunction constitutes a primary defect in obesity and might link this disease to severe chronic health problems. We aimed to evaluate the antioxidant activity of three extracts from *Taraxacum officinale* (dandelion) as well as their effects on mature 3T3-L1 adipocytes concerning intracellular lipid accumulation and cytotoxicity, this would give indications regarding therapeutic interest of dandelion as potential anti-obesity candidate. Antioxidant activities of extracts from dandelion roots and leaves were evaluated *in vitro* using 1,1-diphenyl-2-picrylhyorazyl (DPPH) and Ferric Reducing Antioxidant Power (FRAP) methods at the concentration range used in cellular assays (300–600 µg/mL). The influence of the extracts on mature 3T3-L1 adipocyte viability was determined by the 3-(4,5-dimethylthiazol-2-yl)-2,5-diphenyltetrazolium bromide (MTT) assay. Lipid content was determined by Oil-red-O staining. The extracts showed effective antioxidant activity correlating with total flavonoid and polyphenol contents. However, the functionality level was weakly associated with the antioxidant activity. Further, our data demonstrated that mature 3T3-L1 adipocytes reduced in size and number when incubated with the extracts, which suggests a significant increase in lipolysis activity. Particularly, leaf extract and crude powdered root of dandelion reduced triglyceride accumulation in mature 3T3-L1 adipocytes to a greater extent that the extract from the root. Our study shows anti-lipogenic effects of dandelion extracts on adipocytes as well as radical scavenging and reducing activity. Importantly, along with previous results indicating that cell populations cultivated in the presence of the dandelion extracts decrease in 3T3-L1 adipogenesis capacity, these results suggests that these extracts might represent a treatment option for obesity-related diseases by affecting different processes during the adipocyte life cycle.

## 1. Introduction

Free radicals play a dual role in the organism as both deleterious and beneficial species. In low/moderate concentrations, free radicals or reactive oxygen species (ROS) are involved in normal physiological functions required for cellular signaling. However, excessive production of free radicals or a decrease in antioxidant levels leads to oxidative stress. Increased oxidative stress has been implicated in the pathogenesis of some chronic diseases associated to aging [1]. Although there is evidence to support the role of dietary antioxidants in the prevention, incidence and severity of cancer, cardiovascular and neurodegenerative diseases [2], randomized trials of antioxidants have generally failed to demonstrate this effect [3,4]. However, it is important to note that antioxidants (mixed with other components) consumed directly with the diet might not be the same as those pure compounds usually used in clinical trials. The two major classes of antioxidants are enzymatic and non-enzymatic. The enzymatic antioxidants are produced endogenously and include superoxide dismutase, catalase and glutathione peroxidase. The non-enzymatic antioxidants are those consumed in our diet and include phytochemicals like tocopherols, carotenoids, ascorbic acid, flavonoids and tannins, most of which are generally obtained from natural plant sources and are consumed mixed. Although epidemiological studies point out that intake of medicinal plants lowers the risks of several chronic diseases, it is not clear whether these results are related to their amount of antioxidants, other components or other healthy lifestyle factors generally associated with the consumption of these products.

Increased oxidative stress in accumulated fat has been linked to metabolic syndrome [5], which points out the modulation of the redox state of the adipose tissue as a therapeutic target to prevent dyslipidemia and obesity. At the cellular level, obesity is characterized by hypertrophy (cell volume/size increase) and hyperplasia (cell number increase) of adipocytes from undifferentiated fibroblast-like preadipocytes. Both processes are largely dependent on the regulation of adipocyte differentiation. Therefore, treatments that regulate volume/size and number of adipocytes might provide a better therapeutic approach to manage obesity. Increasing evidence suggests that the phytochemical constituents of natural plant extracts exert anti-obesity effects by inhibiting preadipocyte differentiation and by attenuating the adipose tissue growth as well as by inducing apoptosis and promoting lipolysis of mature adipocytes [6]. Due to the limited number of pharmacological agents, the current therapeutic strategy for treating obesity is mainly focused on diet control and physical exercise. The use of edible natural plants has raised considerable interest due to their relatively safe profile, low cost and availability. These products exert the potential anti-obesity effects through different mechanisms, including lipid absorption, intake and expenditure of energy, increase of lipolysis, decrease of lipogenesis and differentiation and proliferation of preadipocytes [6]. Moreover, the growing market for novel phytochemicals to treat the pandemic obesity justifies the research of either unusual sources of phytochemicals such as *Taraxacum officinale.*

*Taraxacum officinale*, commonly named dandelion is an edible plant distributed worldwide, which belongs to the *Asteraceae* or *Compositae* (also referred to as the aster, daisy, or sunflower) family, same as vegetables such as artichoke, cardoon, sunflower, lettuce, endive, chicory and scorzonera [7]. Dandelion is considered to be among the most well tolerated medicinal plants, with virtually no documented side effects. The European Scientific Cooperative on Phytotherapy (ESCOP) and the German Commission E authorizes the use of dandelion root for the restoration of liver function. As a food, dandelion is used as a salad ingredient, in deserts, and the roasted root is used as a coffee substitute. Leaves are a rich source of a variety of vitamins and minerals [7]. In particular, dandelion leaves contain more vitamin A than carrots and an amount of potassium of about 297 mg per 100 grams of leaves [8,9]. Chemical constituents present in the dandelion leaves are bitter sesquiterpene lactones principally also known as bitter principles, several polyphenols and coumarins [9]. Flavonoids have also been isolated from the flowers and leaves, and an abundance of sesquiterpene lactones have been found in dandelion root [10]. Other related compounds include β-amyrin, taraxasterol and taraxerol as well as free sterols (sitosterin, stigmasterin, phytosterin). Further constituents include polysaccharides (fructosans, inulin), smaller amounts of pectin, resin and mucilage [11]. Dandelion is considered to be an excellent choleretic and cholagogue. In Chinese, Arabian and Native American traditional medicine it is used to treat a variety of diseases related to liver, inflammation and cancer [11]. For such an ubiquitous herb, well-designed human studies are surprisingly rare. However, numerous *in vitro* and *in vivo* assays have demonstrated its anti-oxidant and anti-inflammatory properties supporting the long history of dandelion as a folk medicine [11]. Recent research indicates that dandelion and its formulations might contribute to prevent obesity and metabolic disorders via attenuation of oxidative and inflammatory processes [12]. Studies in animals report the effects of different dandelion extracts on various cardiovascular disease risk factors such as obesity and hyperlipidemia [13,14]. Indeed, the inhibitory activity of dandelion on the pancreatic lipase enzyme has been demonstrated *in vitro* and *in vivo* indicating the potential of dandelion as an anti-obesity agent with limited side effects [13].

Our previous results have indicated that three selected dandelion extracts from leaves and roots, polyphenols/flavonoid-rich, play a significant positive role during adipogenesis and lipid metabolism [15]. We also demonstrated that their effects might be linked to the regulation of the expression of a number of genes and long non-coding RNA critical in the control of adipogenesis, supporting their therapeutic interest as potential candidates for the preventive obesity treatment [15]. However, whether their biological effects are linked to their antioxidant activity is not known. Thus, the current work was aimed to evaluate the antioxidant activity of these extracts and their effects on mature 3T3-L1 adipocytes concerning intracellular lipid accumulation and cytotoxicity, which would give an indication for therapeutic interest of dandelion as potential anti-obesity candidate.

## 2. Materials and Methods

### 2.1. Materials

Methanol and ethanol were purchased from Scharlab Company (Barcelona, Spain). Chemicals and reagents such as DPPH (2,2-diphenyl-1-picrylhydrazyl) radical, Trolox (6-hydroxy-2,5,7,8-tetramethyl-2-carboxylic acid), gallic acid, ascorbic acid, BHA, linoleic acid, rutin, potassium ferricyanide, sodium acetate, glacial acetic acid, TPTZ (2,4,6-tripyridyl-1,3,5-triazine), FeCl_3_, β-carotene, insulin, dexamethasone (Dex), 3-isobutyl-1-methylxanthine (IBMX), 4-(2-hydroxyethyl)-1-piperazineethanesulfonic acid (HEPES) buffer solution, 3-(4,5-dimethylthiazol-2-yl)-2,5-diphenyltetrazolium bromide (MTT), Oil-Red-O (ORO), ergosterol, cholesterol, sodium dodecylsulfate (SDS), Serum Triglyceride Determination Kit (Catalog Number TR0100, Sigma-Aldrich, St Louis, MO, USA), tris-HCl, deoxycholic acid, ethylenediaminetetraacetic acid (EDTA), ethylene glycol tetra-acetic acid (EGTA), NaF, Na_4_P_2_O_7_, SiO_2_, Igepal CA-630 and Folin & Ciocalteu’s phenol reagent were acquired from Sigma-Aldrich Company (St Louis, MO, USA). A 4% paraformaldehyde solution in PBS was acquired from Santa Cruz Biotechnology, Inc. (Santa Cruz, CA, USA). Dulbecco modified Eagle’s medium (DMEM), phosphate buffered saline (PBS), fetal bovine serum (FBS), glutamine and an antibiotic-antimycotic mixture were obtained from Invitrogen (Carlsbad, CA, USA). The mouse fibroblast preadipocytes 3T3-L1 cell line (Catalog No. CL-173TM) was purchased from the American Type Culture Collection ATCC) (Manassas, VA, USA).

### 2.2. Preparation and Characterization of Dandelion Extracts

Commercial capsules of root powder were purchased from Arkopharma® (Cedex, France). Extracts prepared from dandelion leaves and roots were kindly supplied by Soria Natural Laboratory (Soria, Spain). Root and leaves extracts and the commercial root powder were dissolved separately in sterile H_2_O, and filtered through a 0.45 µm membrane. After this treatment, the extracts were kept in the dark at 4 °C for no longer than one week prior to evaluation of antioxidant activities and functionality. The flavonoid characterization of the extracts was carried out by reversed-phase high performance liquid chromatography (HPLC) (HPLC system, Agilent 1100 Infinity Series LC, Santa Clara, CA, USA) using a 5 µm C18 column (Agilent Eclipse XDB-C18). For this procedure, a 0.5 g dry sample was suspended in 70 mL of methanol (50% *v*/*v*), and the mixture was heated with reflux and filtered with a 0.45 µm membrane. Separation of flavonoids was carried out by gradient elution using methanol/acetic acid 2% (15:85 *v*/*v*) from 0–5 min, methanol/acetic acid 2% (60:40 *v*/*v*) from 6–11 min, and methanol/acetic acid 2% (80:20 *v*/*v*) from 12–15 min. The eluted compounds were monitored by absorption spectroscopy and identified on the basis of UV absorption and retention time as compared with reference standards. Results are expressed in milligrams per gram (mg/g) of dry weigh extract (DWE) (Table 1).

**Table 1:** Principal flavonoids of crude powdered root, leaves and root extracts were characterized and quantified by high performance liquid chromatography (HPLC). Results are expressed as mg flavonoid/g dry weigh extract (DWE) (ND = Not detected).

Flavonoid	Extract		
	Crude powdered root	Leaves	Root
Luteolin-7-*O*-glucoside	ND	1.03	ND
Myricetin	0.20	ND	ND
Chlorogenic acid	0.81	4.36	0.80
Caffeic acid	1.08	18.89	1.43
Vitexin-2-rhamnoside	0.96	1.99	0.09
Isovitexin	ND	0.91	ND
Hesperidin	ND	ND	0.90
Total	3.05	28.32	3.22

### 2.3. Determination of Antioxidant Activity

The antioxidant activity of three ethanolic extracts of the roots and leaves of *Taraxacum officinale* were evaluated *in vitro* at the concentrations range used in cellular assays (300–600 µg/mL).

### 2.4. DPPH Radical Scavenging Assay

The antioxidant activity of the extracts was measured on the basis of the scavenging activity of the stable 1,1-diphenyl-2-picrylhyorazyl (DPPH) free radical according to the already described method with slight modifications. Briefly, about 200 µL of 0.2 µM solution of DPPH in 100% ethanol was added to 50 µL of the sample (extract dissolved in water) solution of varying concentrations (25; 12.5; 6.25; 3.125; 0 mg/mL). The samples were placed in a 96-well plate and the reaction mixtures—in triplicate—were allowed to stand in the dark at room temperature for 15 min. Ethanol served as blank and DPPH in ethanol without the extracts served as positive control. After incubation, the discoloration of the purple color was read at 517 nm using an UV-visible spectrophotometer. A decrease of the DPPH solution absorbance indicated an increase of the DPPH radical scavenging activity, which was calculated as percentage of inhibition (*I*%) according to the equation

(1) $$I\%=\frac{A_{\text{c}}-A_{\text{s}}}{A_{\text{c}}}\times 100$$

where *A*_c_ is the absorbance of the control and *A*_s_ is the absorbance of the sample. Using the percentage scavenging activities at four different concentrations, the antioxidant activity of the samples were determined by the measurement of the effective concentration, which is the concentration (mg/mL) of the samples (extracts dissolved in water) required to scavenge 50% (IC_50_) of the radicals.

### 2.5. Reducing Power Ability (FRAP Assay)

When the ferric 2,4,6-tripyridyl-s-triazine complex (Fe^3+^–TPTZ) is reduced to the ferrous form (Fe^2+^–TPTZ), an intense blue color is developed. The reducing power was investigated by the Fe^3+^-Fe^2+^ transformation in the presence of the extracts. The Fe^2+^ can be monitored by measuring the formation of Perl’s Prussian blue at 700 nm. The method of FRAP assay used was a modified version of that reported by Benzie and Strain [16]. Briefly, FRAP reagent was prepared by mixing 10 volumes of 300 mM acetate buffer (pH 3.4) with one volume of 10 mM TPTZ in 40 mM HCl and with one volume of 20 mM FeCl_3_6H_2_O. A total of 40 µL of properly diluted samples (diluted extracts in distilled water) were added to 150 µL of freshly prepared FRAP reagent in a 96-well plate. The mixture was incubated at 37 °C throughout the reaction. After 15 min, the absorbance was read using a microplate reader at 593 nm against reagent blank. Trolox (6-hydroxy-2,5,7,8-tetramethylchroman-2-carboxylic acid) was used as a standard and the concentrations to make a standard curve were 0–500 µM. FRAP results were expressed in terms of TEAC (Trolox® equivalent antioxidant capacity) that indicates micromoles of Trolox® that have the same antioxidant capacity value of 1.0 g of dry weight extract (µmol TE/g DWE). All of the treatment groups were measured in triplicate. Mean and standard deviation were calculated.

(2) $$\text{TEAC}=\frac{\text{moles Trolox}}{\text{g extract}}=\frac{\text{μmoles Trolox}}{\text{1 L extract}}\times \frac{\text{0.001 L extract}}{\text{X μg extract}}$$

### 2.6. Cell Culture and Differentiation

Mouse fibroblast 3T3-L1 preadipocytes were cultured as previously described [15]. Briefly, cells were cultured in 10% FBS-supplemented Dulbecco's modified Eagle’s medium (DMEM) supplemented with 10% (*v*/*v*) fetal bovine serum, 4-(2-hydroxyethyl)-1-piperazineethanesulfonic acid buffer solution 25 mM, 1% (*v*/*v*) glutamine, and 1% (*v*/*v*) antibiotic-antimycotic mixture at 37 °C in a humidified atmosphere with 5% CO_2_. Two days after reaching confluence, differentiation was initiated by the addition of adipogenic agents (insulin 5 µg/mL, Dex 0.25 µM, and IBMX 0.5 mM) to the culture media DMEM plus 10% FBS. After 3 days, the medium was removed, and the cells were then maintained in DMEM plus 10% FBS medium and 10 µg/mL insulin until use, usually 1–3 days after completion of the differentiation. Oil-Red-O staining of 3T3-L1 adipocytes was performed as previously described [15]. All experiments were performed ten days after differentiation.

### 2.7. Cell Viability

The influence of the *Taraxacum officinale* extracts on mature adipocytes viability was determined by the MTT assay. Tests were performed in 24-well plates. Cells were seeded (350 cells/well) and grown to maturation as described above. Mature adipocytes were incubated with either 0.2% dimethylsulfoxide (DMSO) or the extracts (400–600 mg/mL) for 48 h. A cell viability assay was performed per the manufacturer’s instructions. A total of 50 µL of the MTT solution (5 mg/mL) was added to each well and the plates were incubated for 3 h at 37 °C. After incubation, 200 µL of DMSO was added to the wells followed by gentle shaking to solubilize the formazan dye. Absorbance was read at 560 nm using a microplate reader (Asys UVM340 Biochrom Ltd., Cambridge, UK) to determine the formazan concentration, which is proportional to the number of live cells. Surviving cell fraction was calculated according to the following equation:

(3) $$\text{cell viability (\%)}=\frac{A_{\text{s}}}{A_{\text{c}}}\times 100$$

where *A*_s_ is the absorbance of the sample and *A*_c_ is the absorbance of the control, and the cytotoxicity is expressed as a percentage relative to control cells (without *Taraxacum officinale* extracts supplementation).

### 2.8. Oil-Red-O Staining

The 3T3-L1 preadipocytes were harvested six days after the initiation of differentiation. Then, cells were washed with PBS and fixed with 4% formaldehyde for 30 min. Cells were stained with the ORO working solution (3 mg/mL in 60% (*v*/*v*) isopropyl alcohol) for 1 h at 25 °C and examined by an optical microscope to evaluate lipid accumulation. Lipids were extracted from the cells using isopropyl alcohol for 1 h and quantified using a microplate reader at 510 nm. The results are expressed relative to differentiated control cells.

### 2.9. Measurement of Lipid Accumulation

Lipid content in mature adipocytes was quantified and determined by staining with ORO working solution, and subsequently, the absorbance was measured at 510 nm. In brief, on the sixth day of differentiation, 3T3-L1 adipocytes grown in DMEM supplemented with 10% FBS were treated with different concentrations (400–600 µg/mL) of the *Taraxacum officinale* extracts for 48 h. In parallel, control cells were cultured in the same medium in the absence of extracts. Experiments were performed with at least six replicates per treatment and repeated three times. Cells were washed with PBS and lipids were extracted with chloroform: methanol (2:1) for 24 h at 4 °C. Extracted lipids were dissolved in isopropyl alcohol and the total intracellular triglycerides content was measured using a commercial triglyceride assay (Triglyceride Determination Kit) according to the manufacturer’s recommendations. Proteins were quantified by the Lowry method [17] and total cellular triglyceride (TG) content was normalized to the cellular protein content. Results are expressed as mg TG/mg protein. The experiments carried out to analyze the effect of the extracts on the intracellular cholesterol content of mature adipocytes tested the extracts concentrations of 400 and 600 µg/mL for 48 h [18]. For this, the cells were washed twice with PBS and lysed in a solution of KOH 10% (*v*/*v*). A volume of 500 µL of this lysate was added to a volume of 20 µL of ergosterol at a concentration of 1 mg/mL as internal standard, followed by an extraction with 2.5 mL of chloroform/methanol (2:1) for two hours. The aqueous phase was removed and the solvent of the lipid fraction was evaporated with N_2_ at 40 °C. The resulting sample of the unsaponifiable fraction was removed with 1 mL of 1 M KOH in 95% ethanol (*v*/*v*) and 200 µL of 1 mM BHT (butylated hydroxytoluene) and maintained at 80 °C for 1 h to produce the saponification. The unsaponifiable fraction was extracted three times with 1.0 mL of hexane, and 200 µL of distilled H₂O. Subsequently the organic phase was evaporated in an evaporator of samples with N_2_ at 37 °C. The fraction obtained was stored at −20 °C until analysis by HPLC (HPLC system, Agilent 1200 Infinity Series LC), for which it was re-suspended in 200 µL of isopropanol. The chromatographic separation was performed using a 5 µm reversed phase C18 column (Agilent Eclipse XDB-C18) at 45 °C with an injection volume of 25 µL, using as mobile phase acetonitrile: water (95:5 *v*/*v*) during the first 27 min and absolute methanol at a flow rate of 1.2 and 3.0 mL/min, respectively. During the chromatographic separation the eluent was monitored by UV-vis spectrometer (Detector Diode-array), and the identification of cholesterol and ergosterol was performed by comparison with retention times of pure standards. For quantification of cholesterol a calibration line using serial dilutions of a pure pattern of cholesterol in isopropanol was built. The results are expressed as a percentage of untreated control cells (100%).

### 2.10. Data Analysis

Data are the mean ± SEM (standard error of the mean) of three independent experiments (* *p* < 0.05) compared with control (Statistical analysis was performed using IBM SPSS Statistics 19.0 for Windows (Chicago, IL, USA). Differences were determined using Student’s *t*-test.

## 3. Results

### 3.1. Caffeic and Chlorogenic Acids and Vitexin-2-Rhamnoside Are the Common Phenolic Compounds in the Dandelion Extracts

The three dandelion extracts were analyzed by HPLC following the established procedure described in the methods section in order to quantify the main phenolic components (Table 1). Analysis of the extracts’ composition showed a wide variety of active phenolic phytochemicals, which greatly differ in their concentrations. Leaf extract contains a higher amount of total phenolics—*i.e.*, 28.32 mg/g dry weigh extract (DWE)—than the crude powdered root extract (3.05 mg/g DWE) and the root extract (3.22 mg/g DWE). These values are consistent with those reported elsewhere for other species of the *Asteraceae* family [7,9]. In general, the most common flavonoids are the 7-*O*-glycosides of apigenin and luteolin.

Caffeic and chlorogenic acids along with vitexin-2-rhamnoside are the major phenolic compounds present in the three studied dandelion extracts. In particular, chlorogenic acid and vitexin-2-rhamnoside, which are present at high concentrations in the leaf extract, were significantly less concentrated in the crude powdered root extract and the root extracts. Similar amounts of caffeic acid were found in the three extracts. Interestingly, the amount and type of flavonoids found were remarkably different between both the extracts from roots. In particular, hesperidin was only found in the root extract, whereas myricetin was uniquely detected in the crude powdered root extract. In addition, luteolin-7-*O*-glucoside and isovitexin were present in the leaf extract but absent in the root extracts. The identified phytochemicals in this study were found to be consistent with those found from the other studies for the dandelion plant [19,20].

### 3.2. Effects of Dandelion Extracts on Cell Viability of Mature 3T3-L1 Adipocytes

Prior to determining the functional effects of the extracts on mature adipocyte, their impact on cell viability and survival were evaluated using an MTT assay. To this end, mature 3T3-L1 adipocytes were cultured separately for 48 h with the extracts at different concentrations (400, 500 and 600 µg/mL), which was the range employed in cellular and functional assays. The commercial crude powdered root dose is suggested to be six capsules per day, which is equivalent to 1500 mg/day for humans (according to the manufacturer). Although the intake of this concentration is recommended, the biological effects *in vivo* of all these extracts need to be validated first. The effect on the viability of mature 3T3-L1 adipocytes is shown in Table 2. Interestingly, while the MTT assay in a previous study revealed only subtle changes in preadipocyte viability at all tested concentration of the three dandelion extracts [15], the viability of mature adipocytes at higher concentrations of crude powdered root extract was significantly reduced. Further, this extract reduced the viability of adipocytes in a dose-dependent way, reaching the maximum effect—about 81.90% ± 0.7% of differentiated control cells—at a concentration of 600 µg/mL. The root extract produced no significant effect on cell viability, whereas, surprisingly, the leaf extract caused increased adipocyte viability in a dose-dependent way.

**Table 2:** Effect of dandelion extracts on cell viability, intracellular content of triglycerides (TG) and cholesterol in mature 3T3-L1 adipocytes. Cell viability of mature 3T3-L1 adipocytes after 48 h of treatment with dandelion extracts with the indicated concentrations (µg/mL). Results are expressed as percent viability relative to untreated control positive adipocytes (100%); Intracellular triglyceride content of mature 3T3-L1 adipocytes treated for 48 h with dandelion extracts with the described concentrations (µg/mL) and untreated (positive control) cells. Results are expressed as mg triglycerides/mg protein; Cholesterol content of mature adipocytes treated for 48 h with extracts with the described concentrations (µg/mL). Cholesterol content was analyzed and quantified by high performance liquid chromatography (HPLC). Results are expressed as percentage of cholesterol (mg/mL) compared to differentiated positive control cells (100%). Results are expressed as the mean ± SEM of three independent experiments. Differences between groups compared to untreated control cells (positive control) are calculated considering those significant at *p* < 0.05.

Treatment adipocytes	Cell viability (% of the control)	Triglycerides (mg TGs/mg protein)	Cholesterol (% of the control)
Untreated Adipocytes	100.0 ± 0.4	0.229 ± 0.005	100.0 ± 0.49
Crude powdered root (µg/mL)			
400	92.80 ± 0.6	0,179 ± 0.001	87.109 ± 0.285
500	90.80 ± 0.7	0.178 ± 0.003	79.456 ± 0.564
600	81.90 ± 0.7	0.176 ± 0.004	74.724 ± 0.323
Root extract (µg/mL)			
400	92.8 ± 0.6	0.220 ± 0.005	73.548 ± 0.963
500	96.7 ± 0.2	0.228 ± 0.002	68.521 ± 0.778
600	97.0 ± 0.5	0.239 ± 0.002	60.729 ± 0.486
Leaves extract (µg/mL)			
400	105.5 ± 0.5	0.168 ± 0.001	88.921 ± 0.838
500	107.1 ± 0.5	0.165 ± 0.001	88.128 ± 0.779
600	109.6 ± 0.2	0.150 ± 0.004	87.164 ± 0.874

### 3.3. Dandelion Extracts Reduce Lipid Accumulation in Mature Adipocytes

Lipid accumulation analysis of mature 3T3-L1 adipocytes treated during 48 h with dandelion extracts are shown in Table 2. Dandelion leaf extract and the crude powdered root extract significantly reduce the content of intracellular triglycerides in mature 3T3-L1 adipocytes at the tested doses (Table 2). The leaf extract at a concentration of 600 µg/mL produced the greatest impact reducing triglyceride levels by up to 34% (0.150 ± 0.004 mg TGs/mg protein) compared to control (0.229 ± 0.005 mg TGs/mg protein). Although smaller, at concentrations of 400 and 500 µg/mL, the effects of this extract are also significant, reducing the triglycerides accumulation by up to 26% (0.168 ± 0.001 mg TGs/mg protein) and 28% (0.165 ± 0.001 mg TGs/mg protein), respectively. Moreover, the crude powdered root extract produces a considerable decrease in triglyceride levels by up to 22% compared to control; this effect was observed at all doses. Nevertheless, although slight and non-significant, an unexpected and inexplicable rise in triglyceride levels was observed with root extract supplementation at the tested dose.

Total cholesterol content (Table 2) was also significant reduced with the crude powdered root extract (~25%) at 600 µg/mL. Moreover, the root extract exhibited a higher effect, i.e. reducing the cholesterol content by ~39% at 600 µg/mL. Treatment with dandelion leaf extract resulted in a moderate constant decrease of cholesterol levels at the three tested concentrations. Therefore, treatment with dandelion extracts during two days seems to be critical for lipid accumulation in adipocytes. These results indicate that crude powdered root extract has a potential to act directly on mature adipocytes to reduce cell viability and lipid content (triglycerides and cholesterol), as well as repress adipogenesis as previously demonstrated [15]. Likewise, leaf extract decreases triglycerides to a greater extent, but cholesterol only moderately, and the root extract decreases the cholesterol levels to a greater extent, but not the triglycerides content.

### 3.4. Dandelion Extracts as Potential Source of Natural Antioxidant

As an antioxidant capacity of different parts of *Taraxacum officinale* (reviewed in [11]) might be involved in its biological effects [21,22], we thus evaluated the antioxidant activity of the three extracts using two different methodologies. To this end, the FRAP (Ferric Reducing Antioxidant Power) [16] and the scavenging of free radical DPPH (2,2-diphenyl-1-picryl-hidrazil) methods [23] were used.

As shown in Table 3 the highest DPPH radical scavenging activity (a lower value of EC50 indicates a higher activity) was detected in the leaf extract with an EC50 of 1.9 µg/mL, followed by the root extract with an EC50 of 12.6 µg/mL and the crude powdered root extract with an EC50 of 65.0 µg/mL. This is consistent with the highest total phenol levels. Compared to pure compounds, its antioxidant activity is similar to that of pure polyphenols such as catechin (2.17 ± 0.11 µg/mL), caffeic acid (1.94 ± 0.08 µg/mL) and quercetin (1.63 ± 0.07 µg/mL) [24]. Our results are in agreement with previous activity found for aerial parts of *Taraxacum officinale* using the DPPH method [11,25,26].

**Table 3:** Antioxidant activity of *Taraxacum officinale* extracts determined by Ferric Reducing Antioxidant Power (FRAP) and 2,2-diphenyl-1-picryl-hidrazil (DPPH) methods; Results are expressed as TEAC (Trolox equivalents) in µmol Trolox®/g dry weigh extract (DWE) and as EC_50_ (µg/mL). Data are the mean ± SEM of four independent experiments.

Extracts	FRAP	DPPH
	TEAC (µmoles Trolox®/g)	EC_50_ (µg/mL)
Leaves extract	302.3 ± 26.3	1.9 ± 0.1
Root extract	124.5 ± 14.8	12.6 ± 1.3
Crude powdered root	25.2 ± 4.1	65.0 ± 0.4

FRAP assay measures the reducing potential of an antioxidant and is widely used in the evaluation of antioxidant activity of dietary polyphenols [27]. As also shown in Table 3 the trend of the reducing power of the three extracts was similar to those values obtained by the DPPH assay and consistent with them. The leaf extract still provided the highest antioxidant capacity with a FRAP value of 302.3 ± 26.3 µmol TE/g DWE, indicating a good antioxidant activity in the FRAP assay. The reducing power values for the root and crude powdered root extracts were 124.5 ± 14.8 and 25.2 ± 4.1 µmol TE/g DWE, respectively.

In general, the three extracts showed effective reducing power and free radical scavenging activity. However, based on these results, it is possible to infer that the leaf extract of *Taraxacum officinale*, which is rich in phenolic and flavonoid compounds, not only presented the highest free radical scavenging capacity but also the strongest reducing capacity (Table 3). The results obtained in this study indicate that the leaf extract of *Taraxacum officinale* and, to a lesser degree, the root extract are potential sources of natural antioxidants. Although all extracts exhibited antioxidant activity in the methods used, their antioxidant effects were not directly related to their lipid accumulation effects on mature adipocyte.

## 4. Discussion

For a potential application of dandelion extracts as an anti-obesity therapeutic alternative, it is crucial to understand their biological effects in the cellular environment. Here we showed that dandelion extracts exhibit anti-adipogenic effects on mature adipocyte. Interestingly, we found that this effect was not directly related to the antioxidant activity of the extracts, which suggests that extract functionality can be attributed to diverse mechanisms that do not necessarily involve the antioxidant responses. We have recently reported that dandelion leaf and root extracts—the same extracts as those studied here—inhibited 3T3-L1 adipocyte differentiation by preventing adipocyte lipid accumulation [15]. DNA microarray analysis showed that the extracts regulated the expression of a number of genes and long non-coding RNAs that play a major role in the control of adipogenesis [15]. In particular key genes related to cellular differentiation (GO: 0030154; Dmkn, Tcfap2c, Ndrg2, Peg10, Trp63, Serpine2, Micalcl, Dlk1, Adam18, Ctgf, Nme5, Vegfa and Rorc), brown fat cell differentiation (GO: 0050873; Adrb2, Adrb1, Bnip3 and Slc2a4), and diet-induced thermogenesis (GO: 0002024; Adrb2 and Adrb1) were regulated by the effect of the leaf extract. Based on these results, in this study we investigated the same dandelion extracts in order to study their impact on lipid accumulation within already mature 3T3-L1 adipocytes. Our data herein suggest that these extracts also contribute to normalize cholesterol and triglycerides levels, thereby improving the lipid profile of mature adipocytes. Moreover, our preliminary studies suggest that dandelion extracts might reduce the expression of key genes involved in 3T3-L1 mature adipocyte biology, including Pparγ (peroxisome proliferator-activated receptor γ), Adipoq (Adiponectin) and Mapkap1 (mitogen-activated protein kinase associated protein 1), and through this, exert in part their biological effects. However, we do not discard other additional mechanisms to be involved, including long non-coding RNAs, which might play a role.

According to our chromatography analysis, the most representative phenolic/flavonoids compounds present at high concentrations in the three investigated extracts were caffeic and chlorogenic acids along with vitexin-2-rhamnoside (apigenin). In addition, hesperidin was merely found in the root extract; myricetin was uniquely detected in the crude powdered root extract; and luteolin-7-*O*-glucoside and isovitexin were both present in the leaf extract. Most of the literature references vitexin-2-*O*-rhamnoside, an apigenin flavonoid, to be extracted from hawthorn leaf extract, which has been widely used for treating cardiovascular and fatty liver diseases [28]. Chlorogenic and caffeic acids are the major components of our extracts, which are hydroxycinnamates [29] with a wide variety of biological properties. Recently, the roles and applications of chlorogenic acid, particularly in relation to glucose and lipid metabolism, have been highlighted [30]. Both chlorogenic and caffeic acids possess antioxidant properties *in vivo* [31,32] and have been reported to prevent different cancers and cardiovascular diseases in animal models [33].

Our results indicate that the leaf extract, which has the highest total phenolic content, also exerts the best antioxidant activity as evaluated by the two assays. Unexpectedly, the antioxidant capacities values of the two extracts obtained from the roots are remarkable different (Table 3), despite a similar total phenolic composition (Table 1). Indeed, dandelion root extract showed a higher antioxidant activity than the crude powdered root extract. It is likely that the different phenolic composition might be responsible for this effect, as the root extract contains hesperidin and the powdered crude root has myricetin. However, we do not discard the possibility that other components (not evaluated in this work) or the extraction procedure are responsible for this effect, as myricetin and hesperidin are strong potent antioxidants with similar antioxidant effects [34]. Myricetin, a component of the crude powdered root, also has hypolipidemic and anti-inflammatory properties [35]. Neither do we discard mutual interactions between the diverse components of the extracts or between phenolic compounds present in each extract. Other chemical constituents present in the crude powdered root extract like sesquiterpene lactones, steroids, terpenoids and tannins might influence their activity. Numerous studies have shown that in food synergetic or antagonistic interactions occur between antioxidants resulting in a higher or lesser antioxidant capacity than individually isolated antioxidants. Apart from this, we found that although the antioxidant activity correlates with the amount of phenols/flavonoids of the extracts, the functionality level is insignificantly or weakly correlated with it. This fact is also observed during adipogenesis when 3T3-L1 cells are treated with the same extracts as we have recently shown [15].

Concerning the MMT results, we do not discard the possibility that the increment in MTT results for leaf extracts might be associated with increased mitochondrial activity or the number of mitochondria in differentiated cells but not with increased cell numbers [36]. This suggests that leaf extracts might be an activator of mitochondrial metabolism.

Concerning lipid accumulation, our results suggest that extract functionality can be attributed to diverse mechanisms that might not necessarily involve antioxidant responses. Indeed, supplementation of mature 3T3-L1 adipocytes with crude powdered root extract—with moderated antioxidant capacity—produces a significant decrease in intracellular triglycerides and total cholesterol levels. It also seems to reduce the size/volume and the number of mature adipocytes, which is accompanied by a reduced viability in a dose-dependent manner. The reduced adipocytes viability leads to dramatic hypertrophy of the remaining cells and the appearance of a population of small adipocyte-like cells. We speculate that hypertrophic cells accumulate excessive amounts of lipid as compensation and that these cells ultimately could be more susceptible to apoptosis. Furthermore, although the root extract does not seem to reduce the level of triglycerides, it lowers the cholesterol content in mature adipocytes at the highest concentrations tested (500 and 600 µg/mL). Moreover, in addition to being the extract with the highest amount of phenols and antioxidant capacity, supplementation of 3T3-L1 cells with dandelion leaf extract, produces an increase in the viability of adipocytes, suggesting its action as an activator of mitochondrial metabolism. Supplementation of 3T3-L1 cells with dandelion leaf extract does not affect cholesterol levels at a concentration of 400 µg/mL, the lowest concentration tested. However, at 500 and 600 µg/mL, the decrease is constant. The presence of leaf extract reduced the level of triglycerides and size/volume of mature adipocytes by 30% with all tested concentrations compared to control cells. These hypolipidemic effects could be attributed mainly to the presence of luteolin-7-*O*-glucoside and isovitexin as reviewed in [6]. Isovitexin, a naturally occurring flavonoid contained in dietary rice products [37] with known marked antioxidant and anti-inflammatory properties [37], is a molecule with potent anti-diabetic activity in a murine model of type 2 diabetes [38].

Changes in cellular triglyceride levels were not necessarily accompanied by cholesterol levels in all extracts, *i.e.*, leaf extract dramatically reduced the triglyceride content but cholesterol changes were modest. It is not well characterized how triglyceride levels modify cholesterol content in our model. It is important to note that dyslipidemia in general dramatically contributes to obesity. In the adipocytes for instance, an inability to further accumulate lipids leads to inflammatory processes, which induce macrophage infiltration and cytokine release, which worsen the phenotype [6]. Thus, defining the interaction between triglycerides and intracellular cholesterol pools is critical for a full understanding of the crucial role of triglycerides in the evaluation and management of cardiovascular disease risk. As several natural compounds might act in different ways on the complex biological pathways of adipose biology, it is reasonable to treat adipocytes with a combination of dietary bioactive components. This approach might exceed the favorable effects of each individual compound and lead to additive or synergistic effects on different stages of the adipocyte life cycle. However, we have also to consider the bioavailability of these components, as several polyphenols are rapidly metabolized and their final concentration in tissues might not be high enough to exert a biological effect on their own. In summary, dandelion extracts, which seem to have an inhibitory effect on lipid accumulation during adipocyte differentiation and display different impacts on the expression of relevant genes, also normalize cholesterol and triglycerides levels in mature 3T3-L1 adipocytes, thereby improving lipid profiles. Taken together, these results suggest that *Taraxacum officinale* components might have applications in prevention and treatments of obesity. The relevance of our findings is that we could benefit from the effects of dandelion by dietary supplementation. Unlike conventional drugs, wild dandelion has low a cost and is easy to grow and harmless, so daily consumption could reach adequate doses to exert a biological effect. However, appropriate *in vivo* studies need to be performed in order to validate our promising results.

## 5. Conclusions and Future Directions

Due to the global increase in obesity, identifying natural compounds that inhibit the formation of new adipocytes and prevent the accumulation of fat at different stages of the adipocyte life cycle is a major challenge of biomedical research. In accordance, we have recently demonstrated that dandelion extracts partially inhibit the adipogenesis process [15] and reduce the accumulation of fat in differentiated mature adipocytes (present study). These natural extracts can be used as an easily accessible source of natural antioxidants with clear effects on adipocyte biology. Our global increase of obesity reinforces the search for alternative treatments for this pandemic. In this context our finding might have applications in the prevention and/or treatment of obesity. In conclusion, dandelion components play a significant role in the control of lipid metabolism and adipogenesis, effects that are independent of antioxidant activity. The precise mechanism of their biological effects and their anti-obesity effects *in vivo* deserve further investigation.
